# Evaluation of a Pharmacist Led Oral Chemotherapy Clinic: A Pilot Program in the Gastrointestinal Oncology Clinic at an Academic Medical Center

**DOI:** 10.3390/pharmacy8010046

**Published:** 2020-03-20

**Authors:** Julianne O. Darling, Farah Raheem, Katelyn C. Carter, Elizabeth Ledbetter, Jennifer F. Lowe, Christopher Lowe

**Affiliations:** Health Simon Cancer Center, Indiana University, Indianapolis, IN 46202, USA; Fraheem@IUHealth.org (F.R.); KCarter25@IUhealth.org (K.C.C.); eledbetter@IUhealth.org (E.L.); JLowe3@IUHealth.org (J.F.L.); CLowe@IUhealth.org (C.L.)

**Keywords:** oral chemotherapy, oral oncolytic, oncology pharmacy, education, patient satisfaction, gastrointestinal oncology, specialty pharmacy

## Abstract

Oral chemotherapy represents a major patient-centric advancement in therapy convenience. However, ownership of safe and correct administration of these agents requires significant patient education. To address this challenge, an in-person pharmacist-led oral chemotherapy education clinic in gastrointestinal oncology patients within an academic medical center was created and assessed. In this pilot program, a medication-specific quiz was administered to patients before and after education performed by a pharmacist to assess patient understanding of their new oral chemotherapy. A five-question satisfaction survey was also administered at the conclusion of the pharmacist clinic visit. Primary outcome was the percentage difference between pre-and post-education quiz scores. Secondary outcomes included patient satisfaction, time to treatment initiation, and number of pharmacist interventions. Frequencies and medians were used to describe categorical and continuous variables, respectively. Of the 18 patients analyzed, 50% were male and median age was 59.5 years. Approximately 28% had colon cancer, and 61% were treated with capecitabine. The median post-education scores improved from a pre-education score of 75% to 100%. Overall, seventeen of the eighteen patients responded with “strongly agree” to all satisfaction survey statements. An in-person oncology pharmacist-led oral chemotherapy education session demonstrated an improvement in patients’ understanding of their new oral chemotherapy treatment.

## 1. Introduction

Oral chemotherapy agents have been used in the treatment of cancer for decades. However, it has not been until recently that the market has seen a dramatic expansion in the number of oral chemotherapy agents approved and incorporated into clinical practice guidelines. The use of these agents in clinical practice is an appealing treatment option for patients due to enhanced convenience when compared with intravenous agents.

While oral chemotherapy is more convenient, the use of these agents poses several challenges. There is an inevitable challenge with ensuring adherence to the regimen, safe administration, as well as appropriate storage and handling methods when these tasks are completed by the patients and/or their caregiver. Additionally, many of these agents require complex dosing schedules, as some are only indicated to be taken on specific days of a cycle, and it is the patient’s responsibility to follow the regimen accurately. Adherence to necessary lab monitoring is another challenge. Generally, administration of intravenous chemotherapy is preceded by appropriate laboratory monitoring. Prescribers of oral chemotherapy rely on patients to be compliant with obtaining necessary laboratory tests.

Due to these inherent difficulties, thorough patient education is of utmost importance to ensure accurate administration and adherence. While education can be performed via telephone for convenience, its success can be limited by various factors including, though not limited to, environmental distractions, reliable phone service, and the ability to reach the patient by phone. These factors can also lead to delays in treatment initiation if clinicians must make multiple phone calls to obtain necessary information such as insurance data, financial documents, and signatures on financial assistance paperwork. In-person educations can eliminate some of these limitations, as environmental distractions can be minimized and when scheduled, the provider is guaranteed the ability to reach the patient. This scheduled visit bypasses the inefficiencies caused by missed calls/messages, missing signatures, paperwork, and documentation and avoids a reliance on fax, email, or the postal service. Additionally, the provider can observe the patient’s body language which can provide additional insight into the patient’s level of comprehension. In-person education also provides an opportunity to give patients printed educational materials, medication calendars and adherence tools, as well as the potential to utilize demonstration kits as available to reinforce the information.

In fact, there are several published reports of pharmacist-led oral chemotherapy management programs created to utilize these concepts. Patel, et al. published their study of a pharmacist-led oral chemotherapy monitoring program in prostate cancer patients and reported a significant increase in interventions, lab adherence, and overall duration of time patients spent on each therapy [1]. Lam and Cheung evaluated an oncology pharmacist-managed oral anticancer therapy program for patients with chronic myeloid leukemia and reported higher adherence rates compared to usual care and approximately ten interventions per patient [2]. Muluneh et al. published their results including increased treatment comprehension, adherence, and interventions [3]. Given these positive findings, a pilot program was created to evaluate the effectiveness of a pharmacist-led oral chemotherapy clinic, which aimed to provide in-person educations to patients with gastrointestinal (GI) malignancies starting an oral chemotherapy agent.

## 2. Materials and Methods

Starting in October 2019, pharmacists on the Advanced Therapies Pharmacy (ATP) team at Indiana University Health Simon Cancer Center scheduled GI oncology patients starting a new oral chemotherapy treatment for a 45-minute, face-to-face education visit with an oncology pharmacist. All subjects gave their authorization of consent for inclusion before they participated in the study. The study was conducted in accordance with the Declaration of Helsinki, and the protocol was approved by the Institutional Review Board (IRB).

At the beginning of the education visits, patients were introduced to the pilot program and were informed that their participation, quiz scores, and survey results would be collected as data. Next, they were introduced to a four-question drug-specific quiz (see Appendix A). Questions on this quiz varied from proper administration technique to side effect management and dosing. The medication quiz was utilized to compare the difference between quiz scores prior to their education session, when they would have only previously received education from their physician, and their scores after education with a pharmacist. After the patients completed the pre-session quiz, the pharmacist reviewed important counseling points about the medication including but not limited to, proper administration technique, dosing schedule, storage and handling, common side effects and management, and a review of any drug interactions identified during medication reconciliation. The teach-back method was utilized to ensure that the patient properly understood all important counseling points throughout the education session. After all of the patient’s questions were answered, the patient repeated the same four question quiz to assess comprehension. After reviewing the quiz with the patient, the patient was asked to participate in a five-statement satisfaction survey to gain understanding of the usefulness of the counseling session. This survey was completed in private prior to leaving clinic and given back to the pharmacist in an envelope to minimize potential response bias. Responses to survey statements were designed as a score range of 1–4 with 1 being “strongly agree” and 4 being “strongly disagree.” The highest satisfaction score that can be achieved if a patient responded with “strongly agree” to every statement would be a score of 5. See Appendix B for details on survey statements and scoring system. Our goal was to have a score of ≤2 on each question to indicate that patients found the visit to be helpful overall.

The primary outcome of this pilot program was percentage difference between pre-and post-education quiz scores. Secondary outcomes included patient satisfaction, time to treatment initiation, and number of pharmacist interventions. Frequencies and medians were used to describe categorical and continuous variables, respectively. Mode was utilized to assess patient satisfaction survey questionnaires.

## 3. Results

Between November 2019 and January 2020, 18 patients were seen by an oncology pharmcist in the oral chemotherapy clinic and were included in this pilot program. The median age was 59.5 years (range, 36 to 76 years) with males representing 50% of patients. The most common GI malignancy was colon cancer (28%) followed by rectal cancer and hepatocellular carcinoma (HCC), 22% each. The baseline characteristics of all patients are shown in Table 1.

**Figure 1:**
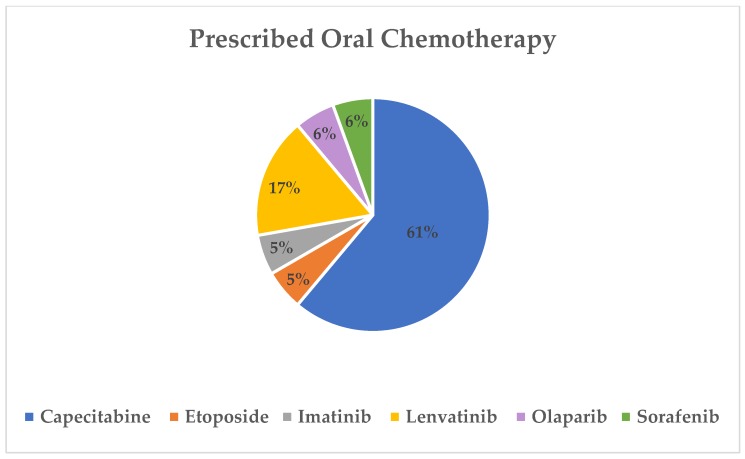
Newly prescribed oral chemotherapy for patients with gastrointestinal (GI) malignancy in the pilot program.

The most commonly prescribed oral chemotherapy agent was capecitabine (61%) followed by lenvatinib (17%). Prescribed oral chemotherapy agents for all patients are shown in Figure 1.

Pre- and post-education quiz questionnaires were administered to all patients. The median pre-education quiz score was 75% (range, 50% to 100%). After receiving pharmacist education during oral chemotherapy clinic visit, the median quiz score increased to 100%.

All patients completed the five-question satisfaction survey administered at the conclusion of their oral chemotherapy clinic visit. Patient satisfaction score mode was 5 (range, 5 to 6). Patient responses to the satisfaction survey are shown in Table 2. Patients responded with “strongly agree” to all survey statements except for statement 1 where one patient responded with “agree.” Overall, 98.9% of patients “strongly agreed” with the satisfaction statements.

The median time to treatment initiaiton was 5.5 days (range, 1 to 29 days). One patient required manufacturer-supplied oral chemotherapy due to high out-of-pocket copay costs, and drug procurement time was 29 days. Half of the oral chemotherapy agents were filled internally at Indiana University Health Advanced Therapies Pharmacy. The remaining prescribed agents were externally filled based on patients’ commercial insurance requirements. A summary of all outcomes is shown in Table 3.

Oncology pharmacists provided at least one medicaton intervention related to the newly prescribed oral chemotherapy in each of the 18 patients. There were a total of 20 pharmacist inteventions in the overall population. The complete list of oncology pharmacist interventions are described in Table 4. The most common pharmacist intervention was identification of drug interactions (40%) followed by identification of baseline tests required prior to initiating specific oral chemotherapy agents (40%).

## 4. Discussion

With the increase in FDA approved indications for oral chemotherapy agents and an emphasis on integrated specialty pharmacy services at academic institutions, oncology pharmacists are in a unique position to positively impact patient care through personalized education sessions during clinic visits. While other studies evaluating the implementation of oral chemotherapy programs exist, this is the first to analyze the impact of in-person pharmacist-led education in patients starting an oral chemotherapy agent for any GI malignancy.

Historically, published data in the GI oncology population has largely focused on capecitabine therapy. In 2007, Macleod et al. reported on their combined nursing and pharmacy oral capecitabine monitoring program in the United Kingdom and reported high patient satisfaction (≥85%) [4]. While in 2011, Simons et al. highlighted increased adherence to capecitabine with a multidisciplinary approach including pharmacists [5]. Hend et al. published the effect of telephone-based follow up on adherence, efficacy, and toxicity of oral capecitabine in May 2019 [6]. Within our institution, telephone-based education and monitoring has been the standard of care since the development of our integrated specialty pharmacy in 2015. While it has been proven that regular follow up with patients on oral chemotherapy has a positive impact on adherence, the limitations of telephone encounters compared to in-person visits for initial patient education has not been explored.

Outside of capecitabine-specific literature, there is no study to date evaluating oral chemotherapy education for all oral therapies in the GI oncology population. With the continually evolving oral treatment options in advanced hepatocellular carcinoma, colorectal cancer, and other GI malignancies, our team recognized the utility of completing a study involving all oral agents utilized in patients with GI malignancies.

The results of our primary outcome suggest that in-person pharmacist education, in addition to the patient’s physician visit, improves patient understanding of their oral chemotherapy. When developing this pilot program, patient satisfaction was a priority for our team. We wanted to ensure patients were receiving detailed and meaningful education sessions without negatively impacting the time they must spend in the cancer center. The GI oncology patient population already spends a significant amount of time coming for follow up visits, lab monitoring, infusions, and potential inpatient admissions. By adding on an additional 45-minute education session, we were concerned we could negatively contribute to their already stressful schedules.

In 2018, Muluneh et al. reported patient satisfaction among patients included in an integrated, closed-loop, pharmacist-led oral chemotherapy program [3]. The authors concluded that 97.8% of the patients in this study reported the teaching provided to them was “good” or “excellent.” While this data is encouraging, we could not extrapolate it to our patient population since we planned for them to return back into clinic for a separate clinic visit specifically with a pharmacist. We worried this added clinic visit could negatively impact patient satisfaction, but our results suggest otherwise. With 98.8% of patients strongly agreeing with the statements in the patient satisfaction survey, we are confident that an added clinic visit to speak with an oncology pharmacist is both beneficial for education and satisfactory to the patient.

Additionally, we found a decrease in time to initiation when patients came back for additional in-person education with an oncology pharmacist. Retrospective data from the GI oncology oral chemotherapy patient population in quarter one of 2019 suggested a turn-around time of 6.5 days. We were able to trim a day off patient time to initiation by having an additional touch point with them in clinic. We speculate that this could be due to the ability to have them fill out any financial assistance paperwork in person instead of waiting to scan, fax, or mail paperwork back and forth between the clinic and the patient.

Furthermore, this program sought to identify aspects of oral chemotherapy treatment where pharmacists made a large impact outside of the education session. In order to do this, we documented any interventions the oncology pharmacist made when chart reviewing a new oral chemotherapy patient. In doing this, we identified multiple drug-drug interactions, missing baseline labs or tests, necessary changes to the medication dosing, and omitted supportive care requirements. Most notably, we found we needed to intervene on baseline monitoring in 40% of the patients included in this pilot program. We have identified this as an area of need for further development, and we plan to discuss collaborative practice agreements to assist with lab monitoring.

This pilot program yields positive results in patient education quiz grades, patient satisfaction scores, and improved time to treatment initiation. However, this is a very small sample size compared to the overall volume of our cancer center. Additionally, we provided the medication education quizzes immediately after the patient received counseling on their new treatment. Moving forward, we hope to continue data collection in a larger volume of patients across multiple malignancies. We also plan to explore alternative times to administer the pre- and post- education quizzes to see if we can determine retention.

## 5. Conclusions

GI oncology patients at our institution showed improved understanding of their new oral chemotherapy after receiving in person education from an oncology pharmacist. These results suggest there is an opportunity to improve patient understanding with the addition of a formalized oral chemotherapy education clinic led by oncology pharmacists.

## Figures and Tables

**Table 1:** Patients baseline characteristics (N = 18).

Characteristic	Value
Age (years) Median (range)	59.5 (36–76)
Sex, male no. (%)	9 (50.0)
GI malignancy diagnosis, no. (%) Appendix adenocarcinoma Carcinoma of unknown primary Cholangiocarcinoma Colon cancer Gastrointestinal stromal tumor Hepatocellular carcinoma Rectal cancer	1 (6.0) 2 (11.0) 1 (6.0) 5 (28.0) 1 (6.0) 4 (22.0) 4 (22.0)

**Table 2:** Patient responses to the satisfaction survey.

Survey Statements	Strongly Agree (%)	Agree (%)	Disagree (%)	Strongly Disagree (%)
Statement 1 ^1^	94.40	5.60	0	0
Statement 2 ^2^	100	0	0	0
Statement 3 ^3^	100	0	0	0
Statement 4 ^4^	100	0	0	0
Statement 5 ^5^	100	0	0	0

^1^ The pharmacist helped me understand why I am taking my new cancer medication. ^2^ The pharmacist made sure that my medicines are safe (possible side effects and avoiding drug interactions). ^3^ The pharmacist helped me understand the best way to take my cancer medication. ^4^ After speaking with my pharmacist, I feel more confident about taking my cancer medication. ^5^ I would recommend my pharmacist to a family member or friend.

**Table 3:** Outcomes of a Pharmacist-Led Oral Chemotherapy Clinic Pilot Program in GI Oncology at Indiana University Health—Simon Cancer Center.

Outcome Measures	Results
Medication-specific quiz scores, median (%) Pre-education Post-education	75 100
Patient satisfaction score, mode (range)	5 (5–6)
Time to treatment initiation in days, median (range)	5.5 (1–29)

**Table 4:** Clinical oncology pharmacist interventions related to the newly-prescribed oral chemotherapy for patients in the pilot program.

Oncology Pharmacist Interventions	Number (%)
Drug interaction identification Baseline monitoring or laboratory test needed Medication dose change Supportive care recommendation Identification of errors during transcription	8 (40.0) 8 (40.0) 2 (10.0) 1 (5.0) 1 (5.0)

## Appendix A

Example of a Pre- and Post-Pharmacist Education Medication Specific Quiz

Capecitabine (Xeloda)

1. How many times a day is capecitabine taken?
  - 1
  - 2
  - 3
  - 4
2. How do you take capecitabine in relation to food/meals?
  - Capecitabine should be taken on an empty stomach
  - Capecitabine should be taken with a snack
  - Capecitabine should be taken within 30 minutes of completing a meal
  - Capecitabine can be taken with or without food, it does not matter
3. Which over the counter medication could you take if you develop diarrhea while on capecitabine?
  - Imodium (loperamide)
  - Pepto-Bismol (bismuth subsalicylate)
  - Prilosec (omeprazole)
  - Metamucil (psyllium fiber)
4. Which of the following ingredients in a skin cream may help prevent Hand-Foot Syndrome caused by capecitabine?
  - Glycerin
  - Hyaluronic acid
  - Aloe
  - Urea

## Appendix B

**Table A1:** Pharmacist-Led Oral Chemotherapy Clinic Patient Satisfaction Survey.

Statement	Score (1–4)
The pharmacist helped me understand why I am taking my new cancer medication.	
The pharmacist made sure that my medicines are safe (knowing possible side effects and avoiding drug interactions).	
The pharmacist helped me understand the best way to take my cancer medication.	
After speaking with my pharmacist, I feel more confident about taking my cancer medication.	
I would recommend my pharmacist to a family member or friend.	

Please rate how you feel about the following statements on a scale of 1 to 4. (1: Strongly agree 2: Agree 3: Disagree 4: Strongly disagree).
